# Anomalous magneto-transport properties of bilayer phosphorene

**DOI:** 10.1038/s41598-020-64106-0

**Published:** 2020-05-06

**Authors:** Jhao-Ying Wu, Wu-Pei Su, Godfrey Gumbs

**Affiliations:** 1 Center of General Studies, National Kaohsiung University of Science and Technology, Kaohsiung, 811 Taiwan; 2 Department of Physics, University of Houston, Houston, Texas USA; 3 Department of Physics and Astronomy, Hunter College at the City University of New York, New York, 10065 USA

**Keywords:** Physics, Condensed-matter physics, Electronic properties and materials, Quantum Hall

## Abstract

The magneto-transport properties of phosphorene are investigated by employing the generalized tight-binding model to calculate the energy bands. For bilayer phosphorene, a composite magnetic and electric field is shown to induce a feature-rich Landau level (LL) spectrum which includes two subgroups of low-lying LLs. The two subgroups possess distinct features in level spacings, quantum numbers, as well as field dependencies. These together lead to anomalous quantum Hall (QH) conductivities which include a well-shape, staircase and composite quantum structures with steps having varying heights and widths. The Fermi energy-magnetic field-Hall conductivity (*E*_*F*_−*B*_*z*_−*σ*_*xy*_) and Fermi energy-electric field-Hall conductivity (*E*_*F*_−*E*_*z*_−*σ*_*xy*_) phase diagrams clearly exhibit oscillatory behaviors and cross-over from integer to half-integer QH effect. The predicted results should be verifiable by magneto-transport measurements in a dual-gated system.

## Introduction

Two-dimensional (2D) layered systems, having nano-scaled thickness and unique geometric symmetries, have found themselves in the main stream of material sciences especially after the success of graphene. These novel materials have been found to possess critical applications due to their transport and optical properties. Other 2D layered structures include silicene^1^, germanene^2^, boron nitride (BN)^3^, gallium nitride (GaN)^4^, transition-metal dichalcogenides (TMDs)^5^, and stanene^6,7^. However, these materials have their own shortcomings. For example, graphene, silicene, germanene and stanene these group IV materials lack significant energy gaps and do not have sufficiently high on-off ratios for electronic applications^8^. TMDs display reasonable on-off ratios, but their carrier mobilities are usually relatively low (mostly lower than 100 cm^2^ *V*^−1^·*s*^−1^)^9^. In other words, the adequate on-off ratio and high carrier mobility hardly appear in these materials simultaneously. On the other hand, V-group elements have been attracting a considerable amount of attention due to their moderate band gap and carrier mobility. Among these elements, few-layer black phosphorus (phosphorene) has been fabricated successfully by using mechanical cleavage^10,11^, liquid exfoliation^12,13^, and mineralizer-assisted short-way transport reaction^14,15^.

The intrinsic energy gap in phosphorene ranges from 0.5 to 2 eV depending on the number of layers^16–18^, as demonstrated by optical measurements^10,19^. Such a gap is larger than that in its bulk counterpart (~0.3 eV)^16,20,21^. Phosphorene-based field-effect transistor has an adequate on/off ratio of 10^5^ and a high carrier mobility of 10^3^ cm^2^/V.s at room temperature^20,22,23^. Additionally, the unusual energy spectra and quantum Hall effect (QHE) due to magnetic quantization^23–32^, make few-layer phosphorene a very good candidate for being a next-generation electronic device^33–35^.

A layer of phosphorene presents a puckered structure owing to the *sp*^3^ hybridization of (3*s*,3*p*_*x*_,3*p*_*y*_,3*p*_*z*_) orbitals. The deformed hexagonal lattice in the x-y plane is a large contrast to the highly symmetric honeycomb lattice of the group-IV systems^36^. This unique geometric structure causes the highly anisotropic low-lying energy bands, i.e., the linear and parabolic dispersions near the Fermi energy *E*_*F*_, respectively, along the $\widehat{{k}_{x}}$ and $\widehat{{k}_{y}}$ directions^17^. The anisotropy clearly reveals in other physical properties, like the recent measurements of the optical and transport properties^20,37,38^. This gives extra advantages for phosphorene in comparison with Mo*S*_2_- and related semiconductors. For example, the unusual anisotropy could be utilized in designing unconventional thermoelectric devices. That is, a thermal gradient and a potential difference could be put on in two orthogonal directions, producing the higher thermal conductivity in one direction and the larger electrical conductivity in another^39,40^. The special intrinsic property will directly reflect in quantization phenomena.

The recent experimental and theoretical researches indicate that graphene and -related systems have rich and unique quantum transport properties. Especially, the magnetic transport measurements of monolayer graphene present the unconventional half-integer Hall conductivity *σ*_*xy*_ = 4(*m* + 1/2)*e*^2^/*h*, where *m* is an integer and the factor of 4 is present due to spin and sublattice degeneracy. The *n* = 0 Landau level (LL), corresponding to the Dirac point, is mainly attributed to the quantum anomaly. For AB-stacked bilayer graphene, the Hall conductivity is in the form of *σ*_*xy*_ = 4*m*′*e*^2^/*h* (*m*′ a non-zero integer). However, an exception is a double step of *σ*_*xy*_ = 8*e*^2^/h at zero energy and low magnetic field^41^. This corresponds to the degeneracy of the *n* = 0 and *n* = 1 LLs of the first group^42^. The unusual phenomena of electronic transport properties of graphene stimulate a lot of interest in exploring the configuration-enriched QHE in other novel 2D materials.

In this paper, we utilize the generalized tight-binding model and the Kubo formula under the linear response approximation to investigate the unusual QHE of bilayer phosphorene. The effects of the geometrical structures, intrinsic interactions, electric field *E*_*z*_ and magnetic field *B*_*z*_ are considered simultaneously. Such method is capable of identifying the magneto-electronic selection rules in the static limit and obtaining the available transition channels in the magneto-transport properties. The relations of quantum conductivity with the Fermi energy *E*_*F*_, *E*_*z*_ and *B*_*z*_ are discussed in detail. They are closely related to the electronic structure, the LLs, and the transition channels.

Changing the electric field strength can greatly diversify the magnetic quantization and enrich the transport properties. The various features could cover half-integer and integer conductivities with distinct steps, a vanishing or non-zero conductivity at the neutral point, and the well-like, staircase, composite, and anomalous plateau structures in the field dependencies. The frequent anticrossing/crossing behaviors of two-groups of LLs may be responsible for the unusual field-dependent characteristics. This illustrates that the feature-rich LLs can create extraordinary magneto-transport properties.

## Methods

**Figure 1:**
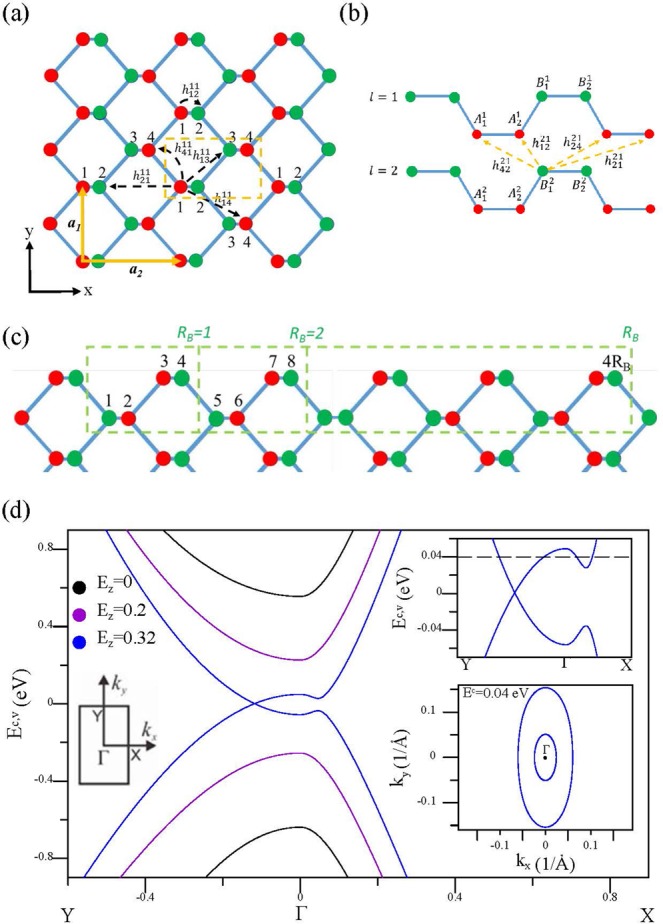
The top view for the geometric structure of monolayer phosphorene in (**a**), and the side view for that of bilayer phosphorene in (**b**), with various intralayer and interlayer atomic interactions. In the presence of a uniform perpendicular magnetic field, an enlarged unit cell is in rectangular shape as shown in (**c**) by the dashed green lines. The band structures of bilayer phosphorene under various electric fields are presented in (d).

Monolayer phosphorene, with a puckered honeycomb structure, has a primitive unit cell containing four phosphorous atoms, as depicted by the dashed yellow lines in Fig. 1(a). Two of the four phosphorous atoms are located on the lower (the red circles) or higher (the green circles) sublattice sites. Similar structures are revealed in few-layer systems, e.g, bilayer phosphorene as shown in Fig. 1(b) for AB stacking. The low-lying energy bands are dominated by the interactions between the 3*p*_*z*_ orbitals^17^. The few-layer Hamiltonian is represented by

1 $$H=\mathop{\sum }\limits_{i\mathrm{=1,}l}^{4}({\varepsilon }_{i}^{l}+{U}_{i}^{l}){c}_{i}^{l}{c}_{i}^{\dagger \,l}+\sum _{\langle i,j\rangle ,l}{h}_{ij}^{ll}{c}_{i}^{l}{c}_{j}^{\dagger \,l}+\sum _{\langle i,j\rangle ,l\ne {l}^{\text{'}}}h{\text{'}}_{ij}^{l{l}^{\text{'}}}{c}_{i}^{l}{c}_{j}^{\dagger \,{l}^{\text{'}}},$$

where *i* and *l* represent the atom and layer numbers, respectively. ${\varepsilon }_{i}^{l}$ is zero in a monolayer, but for a few-layer system, it is a layer- and sublattice-dependent site energy due to the chemical environment. Also, ${U}_{i}^{l}$ is the Coulomb potential energy induced by an electric field. They both contribute to the diagonal matrix elements. In the absence of a magnetic field, the summation is written as ${\Sigma }_{i\mathrm{=1,}l}^{4}$, but in the presence of a magnetic field it becomes ${\Sigma }_{i\mathrm{=1,}l}^{4{R}_{B}}$ (seen later). Also, ${c}_{i}^{l}$ (${c}_{j}^{\dagger \,{l}^{\text{'}}}$) is an annihilation (creation) operator, ${h}_{ij}^{ll}$ and ${h}_{ij}^{\text{'}\,l{l}^{\text{'}}}$ are, respectively, the intralayer and interlayer hopping integrals, and the effective interactions used in the calculations cover the fourth and fifth neighboring atoms. These hopping parameters have been adopted from ref. ^17^.

Black phosphorene is assumed to be in the presence of a uniform perpendicular magnetic field. The magnetic flux through a unit cell is $\Phi ={a}_{1}{a}_{2}{B}_{z}$, where *a*_1_ = 3.27 Å and *a*_2_ = 4.43 Å are lattice constants (the lattice vectors are shown as the yellow arrows in Fig. 1(a)). The vector potential, $\overrightarrow{A}=({B}_{z}x)\hat{y}$, creates an extra magnetic Peierls phase of $\exp \{i[\frac{2\pi }{{\phi }_{0}}\int \overrightarrow{A}\cdot d\overrightarrow{r}]\}$, leading to a new period along $\hat{x}$ and thus an enlarged rectangular unit cell with $4{R}_{B}=4{\phi }_{0}\,/\Phi$ atoms in one layer, as illustrated in Fig. 1(c). Here, *ϕ*_0_ ($=\,h/e=4.1\times {10}^{-15}$ T·*m*^2^) is the magnetic flux quantum; *ϕ*_0_/Φ is chosen to be an integer. The reduced first Brilloun zone has an area of $4{\pi }^{2}\,/{a}_{1}{a}_{2}{R}_{B}$. For bilayer black phosphorous, the magnetic Hamiltonian matrix is very large with 8*R*_*B*_ × 8*R*_*B*_ matrix elements within achievable experimental field strengths, e.g., the dimension of 16800 at *B*_*z*_ = 30 T. The details of the matrix elements can be seen in the Supplementary Information.

The spatial distributions of subenvelope functions derived from the generalized tight-binding model are utilized to characterize the magnetic quantum numbers and the types of LLs^43–45^. They are useful for explaining the peculiar LL behaviors. For example, the quantum number and subgroup classification can be determined by the number of zero points and the dominant sublattices. This enables us to identify the magneto-electronic selection rules in the static limit and obtain the available transition channels in the magnetotransport. The numerically intensive procedure is not restricted to the geometric structure, the number of layers, the stacking order, and the type of external fields. To achieve the experimentally attainable field strengths, a band-like matrix has been developed to solve the huge matrix efficiently^46^. Accordingly, we can observe the strong electrically-tunable LL spectra when *B*_*z*_ < 60 T.

The magnetically quantized LLs can create unique transport properties. Within linear response theory, the transverse Hall conductivity is evaluated from the Kubo formula^47^:

2 $${\sigma }_{xy}=\frac{i{e}^{2}\hslash }{S}\sum _{\alpha \ne \beta }({f}_{\alpha }-{f}_{\beta })\frac{\langle \alpha |{\dot{{\bf{u}}}}_{{\bf{x}}}|\beta \rangle \langle \beta |{\dot{{\bf{u}}}}_{{\bf{y}}}|\alpha \rangle }{{({E}_{\alpha }-{E}_{\beta })}^{2}}\mathrm{}.$$

here, |*α*〉 is a LL state with energy *E*_*α*_, S the area of the enlarged unit cell, *f*_*α*,*β*_ the Fermi-Dirac distribution functions, and ${\dot{{\bf{u}}}}_{{\bf{x}}}$ (${\dot{{\bf{u}}}}_{{\bf{y}}}$) the velocity operator along $\hat{x}$ ($\hat{y}$). The matrix elements of the velocity operators, which determine the permitted inter-LL transitions, are evaluated by the gradient approximation^48^:

3 $$\begin{array}{c}\langle \alpha |{\dot{{\bf{u}}}}_{{\bf{x}}}|\beta \rangle =\frac{1}{\hslash }\langle \alpha |\frac{\partial H}{\partial {k}_{x}}|\beta \rangle ,\\ \langle \alpha |{\dot{{\bf{u}}}}_{{\bf{y}}}|\beta \rangle =\frac{1}{\hslash }\langle \alpha |\frac{\partial H}{\partial {k}_{y}}|\beta \rangle \mathrm{}.\end{array}$$

The above procedure promises the self-conserved number of carriers per area with and without a magnetic field. Since the intra-layer and inter-layer atomic interactions, and the effects of external fields are simultaneously included in the Hamiltonian matrix, the results are accurate and reliable within a wide energy range. The correct LL spectrum and wavefunctions are used for characterizing subgroups of LLs at low energies. The strong competition between them induces the unusual quantization behaviors, such as the frequent anticrossings for the *B*_*z*_- and *E*_*z*_-dependent energy spectra. This brings about the anomalous quantum Hall conductivities. These critical characteristics are either missed or not explained in other papers that use the effective-mass model^49,50^, or consider a one-dimensional ribbon^25,26,31,51^. In ribbons, the quantum confinement would hinder the magnetic quantization leading to partially dispersionless energy spectra^52^ rather than the dispersionless LLs in a two-dimensional system^43–45^. Our method warrants more reliable results on QHE for an arbitrary Fermi level and external field strength.

## Results and discussion

The special lattice structure and complex hopping integrals generate rich band structures. Bilayer black phosphorous (BP) has a direct gap of *E*_*g*_ ≈ 1 eV near the Γ point, as illustrated in Fig. 1(d) by the black curves. The highly anisotropic energy bands yield the approximately linear and parabolic dispersions along ΓX and ΓY (${\hat{k}}_{x}$ and ${\hat{k}}_{y}$), respectively. An electric field *E*_*z*_ could reduce the energy gap considerably. The semiconductor-semimetal transition appears at a strength larger than ${E}_{z,c}\simeq 0.3$ (eV/Å; blue curves), for which the valence and conduction bands are transformed into linearly intersecting bands and oscillatory bands along ΓY and ΓX, respectively, highlighted in the upper inset. The Dirac semimetal state, created by the *E*_*z*_ field, was directly observed by angle-resolved photoemission spectroscopy (ARPES) in bulk^53^ and few-layer BP^54^. The extreme points remain at the Γ point. Two Dirac points (saddle points) are situated on both sides of the Γ point along $+{\hat{k}}_{y}$ and $-{\hat{k}}_{y}$ ($+{\hat{k}}_{x}$ and $-{\hat{k}}_{x}$). Especially, two constant-energy loops, inner and outer, surround the Γ point in the region between the extreme and saddle point energies. This is illustrated by the contour plot for *E*^*c*^ = 0.04 eV in the lower inset. The strongly tunable electronic structure is predicted to bring about peculiar optical^55^ and transport^56^ properties. The electronic states near the Dirac and Γ points are magnetically quantized into two distinct LL subgroups. The hybridization of the two subgroups reaches a maximum at the saddle point energy (${E}^{c}\approx 0.028$ eV; ${E}^{v}\approx -\,0.035$ eV), which could cause anomalous magneto-optical^57^ and -transport properties. It is noted that the *E*_*z*_ effects on the lowest pair of energy bands at the Γ point are qualitatively similar for various layer numbers^58,59^. The different features in the energy bands between them may occur at the other symmetric points and higher energies. This would require the multi-orbital TB calculations^44,60^, for further study.

**Figure 2:**
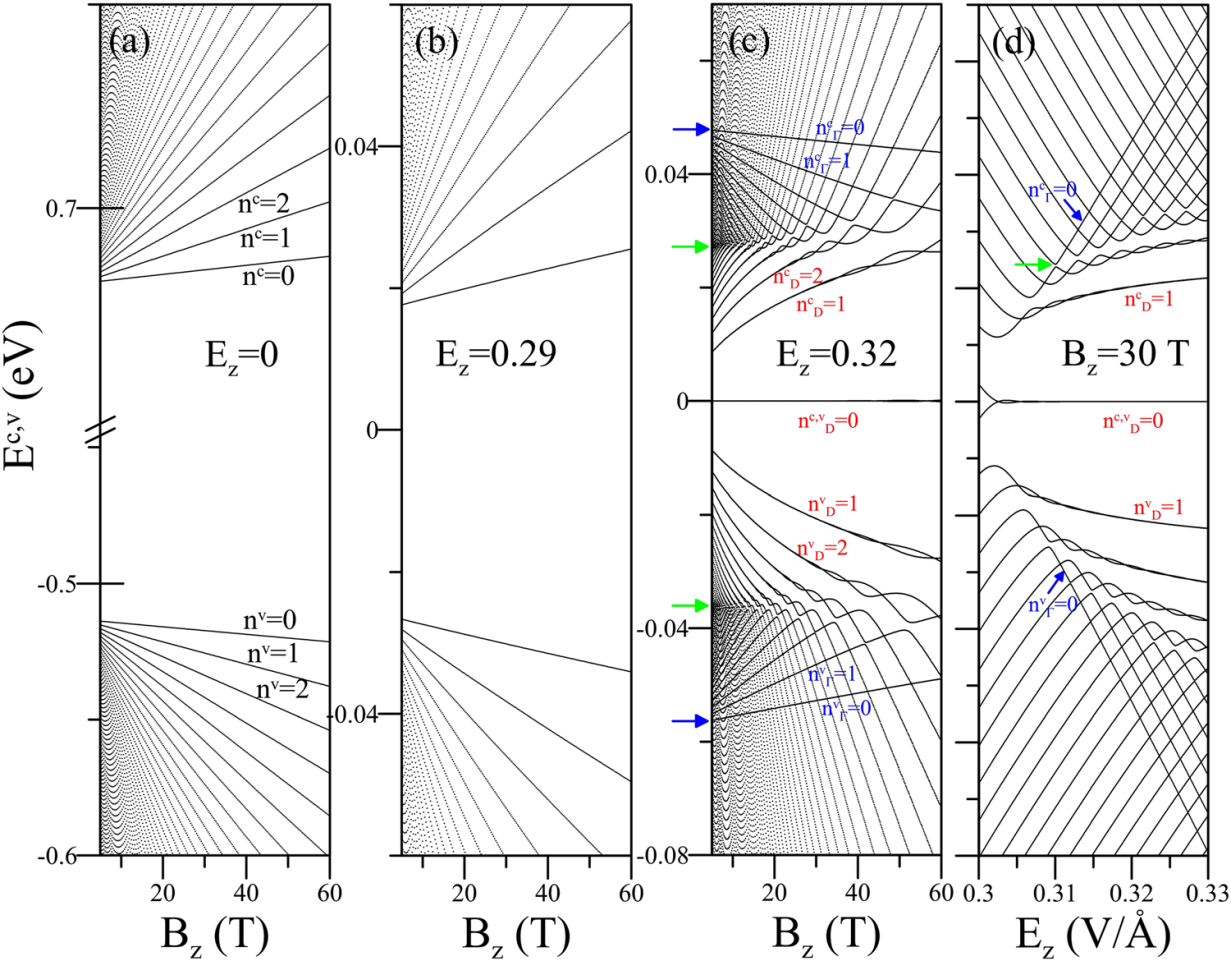
(**a–c**) The *B*_*z*_-dependent LL spectra for various *E*_*z*_’s and (**d**) the *E*_*z*_-dependent LL spectrum at *B*_*z*_ = 30 T.

All the critical points and constant-energy loops in the energy-wave vector space will dominate the main features of the LLs. For ${E}_{z} < {E}_{z,c}$ (Fig. 2(a,b)), the states around the Γ point contribute to the low-lying LLs, which are four-fold degenerate for each (${k}_{x},{k}_{y}$) state including spin and localization-center degeneracies. The occupied LLs are not symmetrically located with respect to the unoccupied ones near the Fermi level. The quantum number *n*^*c*^ (*n*^*v*^) for each conduction (valence) LL is clearly identified from the number of zero nodes in the wavefunctions. For example, the four low-lying conduction/valence LLs have *n*^*c,v*^ = 0, 1, 2; 3. Though they have well-behaved *B*_*z*_-dependence, their energies cannot be well described by a simple relation especially for higher energy and field strength. This is different from the square-root dependence in monolayer graphene^61^, and the linear dependence in AB-stacked bilayer graphene^42^ and *MoS*_2_^62^, due to the highly anisotropic energy dispersion.

A modest value for *E*_*z*_ would lower (raise) the lowest unoccupied LL (highest occupied LL) energy, as shown in Fig. 2(b). The drastic changes in the LL spectrum come about when *E*_*z*_ is increased to over *E*_*z*,*c*_ (Fig. 2(c)). First, the lowest unoccupied and the highest occupied LLs merge together due to the zero band gap. Then, the low-lying LLs evolve into two groups around *E*_*F*_. The lower one, namely the D-group with double degeneracy, could be fitted by a square-root relation $\sqrt{{n}_{D}{B}_{z}}$. That corresponds to the magnetic quantization of electronic states near the two Dirac points, similar to graphene^61^. For the higher one, the Γ-group, beginning at the band-edge-state energies at Γ point (indicated by the blue arrows), the level energies are inversely proportional to *E*_*z*_. Γ-group LLs with their own set of quantum numbers *n*_Γ_ are associated with the inner constant-energy loops (see the lower inset of Fig. 1(d)) and frequently cross the LLs from the outer loops. Γ- and D-group LLs have strong hybridizations around the saddle point energies, indicated by the green arrows, where they cross and anticross each other alternatively. The anticrossings clearly illustrate that such LLs are composed of multi-oscillation modes^43,63,64^. It is worth noting that the two subgroups of LLs will finally evolve into one at the larger *B*_*z*_’s, as shown in the Supplementary Information.

The strong electrically-tunable LL spectrum is demonstrated in Fig. 2(d) for *B*_*z*_ = 30 T. The zero-energy LL is generated at .. The larger *n*^*c,v*^ requires the stronger *E*_*z*_ to form the D-group LL. That is, two entangled LLs gradually merge together with increasing *E*_*z*_. Above the region, one sees that Γ-group LLs are formed beyond the turning points (the first turning point is indicated by the green arrow). The energies of Γ-group LLs increase with *E*_*z*_ quickly. This distinguishes them from the other LLs coming from the outer constant-energy loops. Clearly, the feature-rich LL spectrum strongly depends on the energy regime as well as strengths of the external field. This could result in interesting magneto-transport properties.

**Figure 3:**
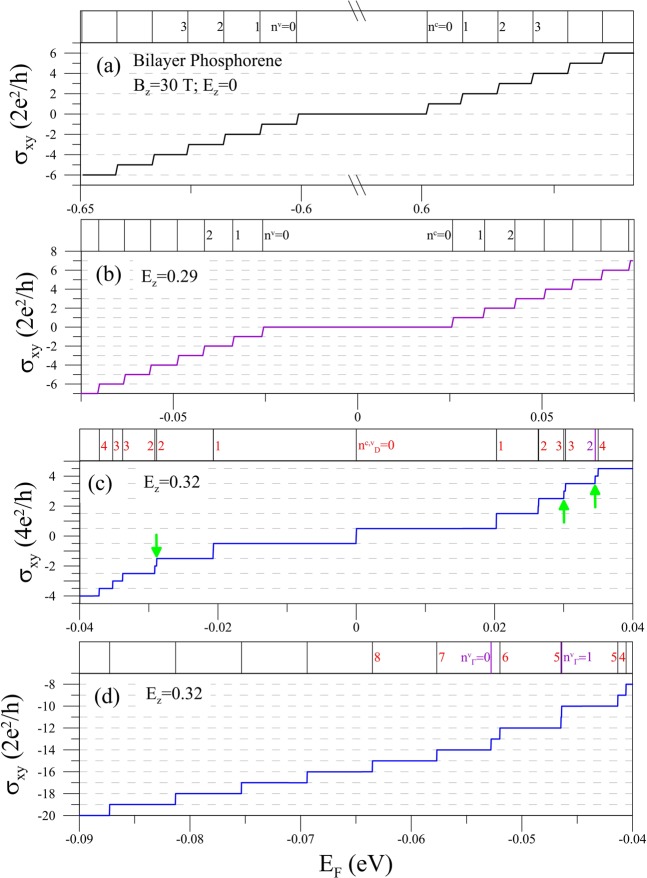
The *E*_*F*_-dependent transverse conductivity at *B*_*z*_ = 30 T for (**a**) *E*_*z*_ = 0, (**b**) *E*_*z*_ = 0.29, and (**c**) *E*_*z*_ = 0.32 eV/Å. Also shown in (**d**) is the lower energy region for *E*_*z*_ = 0.32 eV/Å. The upper inset in each panel shows the corresponding LL energies.

The dependence of the Hall conductivity on the Fermi energy is very sensitive to *E*_*z*_, as exhibited in Fig. 3(a) through 3(d) for *B*_*z*_ = 30 T. When *E*_*z*_ = 0 (Fig. 3(a)), the conductivity is quantized as ${\sigma }_{xy}=2m{e}^{2}/h$ (where *m* is an integer) with almost equal plateau widths. This reflects the parabolic energy dispersion at *B*_*z*_ = 0. For the intrinsic state, only interband inter-LL transitions are allowed. The peak intensities and selection rules for the intrinsic magneto-optical spectra are strongly anisotropic^57^. That is, the selection rules for the x- and y-polarizations are Δ*n* = odd and even numbers, respectively. Since the transverse electric conductivity *σ*_*xy*_ is related to the product of velocity matrix elements along the two directions (Eq. (2)), it becomes zero with the distinct selection rules, leading to a wide zero plateau centering around zero energy. This is different from the case in graphene, where the available interband inter-LL transition channels Δ*n* = 1 and Δ*n* = −1, respectively, possess opposite contributions to *σ*_*xy*_ and cancel each other^65^. The intraband inter-LL transitions determine the magnitude of *σ*_*xy*_. That is, when *E*_*F*_ is between the *n*^*c*^ = *n* − 1 and *n*^*c*^ = *n* LLs (*n* is a positive integer), the transition between the two LLs mainly contributes to *σ*_*xy*_ = + *n* (in units of 2*e*^2^/*h*), while *E*_*F*_ between *n*^*v*^ = *n* − 1 and *n*^*v*^ = *n* LLs it leads to *σ*_*xy*_ = −*n*. Thus, varying *E*_*F*_/passing the LLs (shown in the upper inset) makes the step structures. The width of the zero-conductivity plateau can be reduced by *E*_*z*_ considerably, while for a modest value of *E*_*z*_ the quantization relation *σ*_*xy*_ = 2*me*^2^/*h* remains (Fig. 3(b)). It is until *E*_*z*_ > *E*_*z,c*_ that *σ*_*xy*_ displays distinct features, like in Fig. 3(c) for *E*_*z*_ = 0.32. There are three main characteristics. First of all, the plateau of *σ*_*xy*_ = 0 disappears, which is attributed to the creation of zero-energy LL. Secondly, around *E*_*F*_ = 0 the quantized values of the Hall conductivity are changed from integer to half-integer with the double level degeneracy, i.e., from *σ*_*xy*_ = 2*me*^2^/*h* to *σ*_*xy*_ = 4(*m*′ + 1/2)*e*^2^/*h* (*m*′ a nonzero integer). This manifests the formation of D-group LLs. Thirdly, there exist a few of very narrow plateaus, as indicated by the green arrows. They arise from the lift of LL degeneracy or the appearances of Γ-group LLs (the purple line in the upper inset). Figure 3(d) displays the region of further lower *E*_*F*_. It demonstrates that the coexistence of two groups of LLs could induce the nonuniform plateau widths. However, when *E*_*F*_ becomes smaller than the extreme-point energy at Γ point (≈ −0.053 eV), the structures turn monotonous, i.e, they obey the relation *σ*_*xy*_ = 2*me*^2^/*h*. The regular quantization means that the LLs originating from the outer constant-energy loops (see the lower inset of Fig. 1(d)) become dominant.

**Figure 4:**
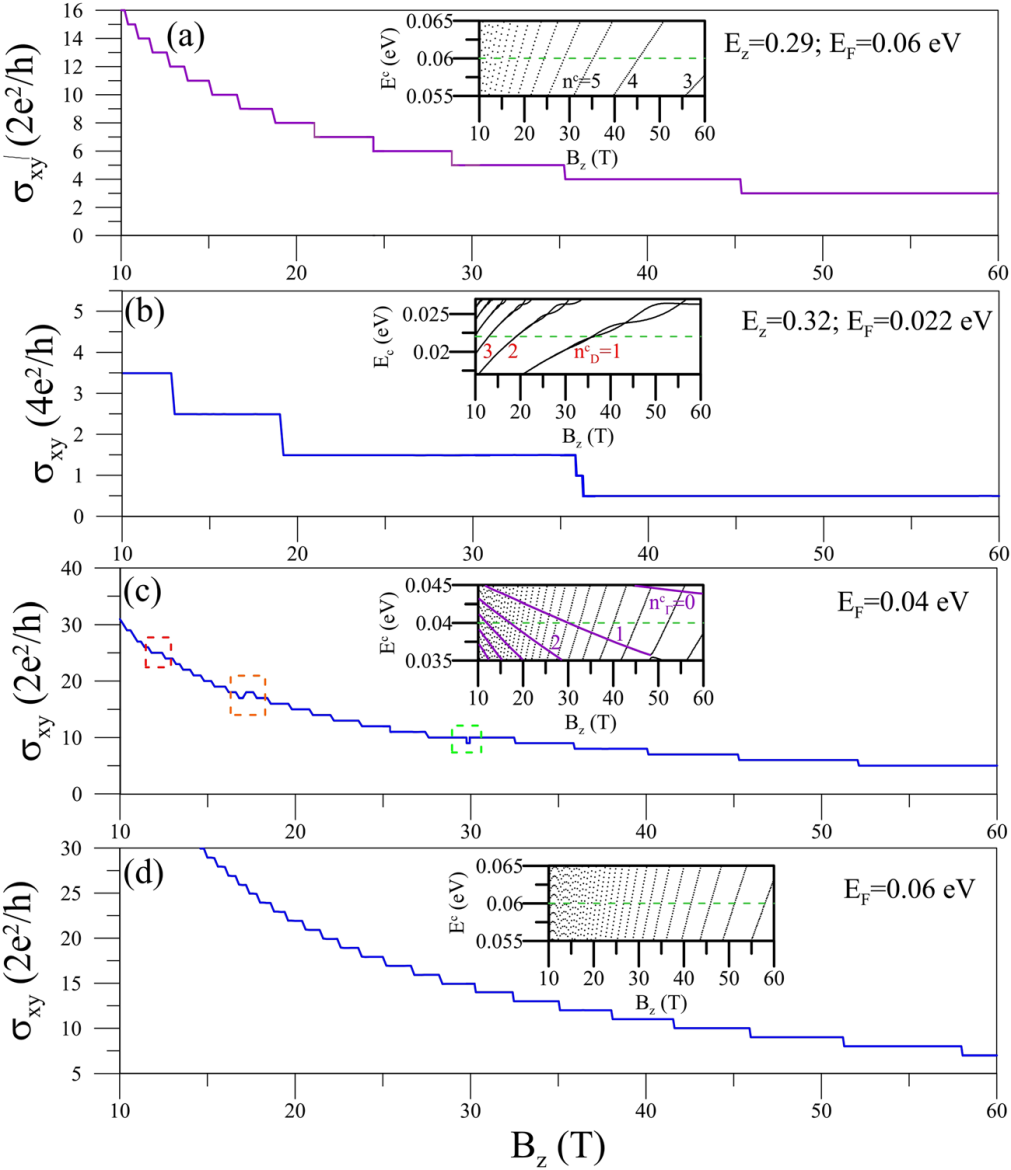
(**a**) The *B*_*z*_-dependent transverse conductivity at *E*_*z*_ = 0.29 eV/Å and *E*_*F*_ = 0.06 eV. The same plots at *E*_*z*_ = 0.32 eV/Å for various *E*_*F*_’s are shown in (**b–d**). Also shown in the insets are the *B*_*z*_-dependent LL energy spectra, with the Fermi levels represented by the dashed green lines.

The *B*_*z*_-dependent Hall conductivity exhibits a variety of features when *E*_*z*_ is varied, as shown in Fig. 4(a–d). These characteristics are due to the behavior of the LL energy spectrum as a function of magnetic field seen in the insets of each panels. For *E*_*z*_ < *E*_*z,c*_ (Fig. 4(a)), strengthening *B*_*z*_ decreases the quantum number of the highest occupied LL and increases the inter-LL spacing of the dominant transition channel around *E*_*F*_ (the dashed green line). Consequently, the height/width of the Hall plateau is reduced/broadened when passing through a LL. This is in marked contrast to the case shown in Fig. 3(a,b) where varying *E*_*F*_ barely changes the plateau width. The value of *σ*_*xy*_ drops to zero over a critical strength *B*_*z,c*_ (not shown). The larger the value of *E*_*F*_ is, the stronger strength of *B*_*z,c*_ is required. As for *E*_*z*_ > *E*_*z,c*_, we observe that there are three main types of *σ*_*xy*_-*B*_*z*_ dependencies for chosen *E*_*F*_, as displayed in Fig. 4(b) through 4(d). Firstly, when *E*_*F*_ is located in the linear energy region (the inset in Fig. 4(b)), the conductivity exhibits the half-integer QHE in units of 4*e*^2^/*h*. There is a narrow plateau at *B*_*z*_ ≈ 36 T due to a pair of entangled ${n}_{D}^{c}=1$ LLs. *σ*_*xy*_ does not achieve the zero value because of the existence of a zero energy LL. Secondly, *E*_*F*_ between in the energies of extreme and saddle points as shown in the inset of Fig. 4(c), where two groups of LLs coexist, the conductivity possesses a composite quantum structure. The reason of this is that the intra inter-LL transitions of D- and Γ-group LLs contribute to positive and negative *σ*_*xy*_, respectively. Therefore, with decreased quantum numbers of D-/Γ-group LLs, the value of *σ*_*xy*_ is reduced/increased. This results in the unusual structures for certain strengths of *B*_*z*_, including the double-width plateau (the red rectangle), bulging (the yellow rectangle) and dip (the green rectangle) structures. The first kind of structure happens when *E*_*F*_ is located at a LL crossing point exactly, while the latter two occur when *E*_*F*_ is slightly above and below a LL crossing point, respectively. Lastly, when *E*_*F*_ is higher than the band-edge-state energy at the Γ point (Fig. 4(d)), the monotonic staircase structure of integer QHE is similar to that of a 2D electron gas.

**Figure 5:**
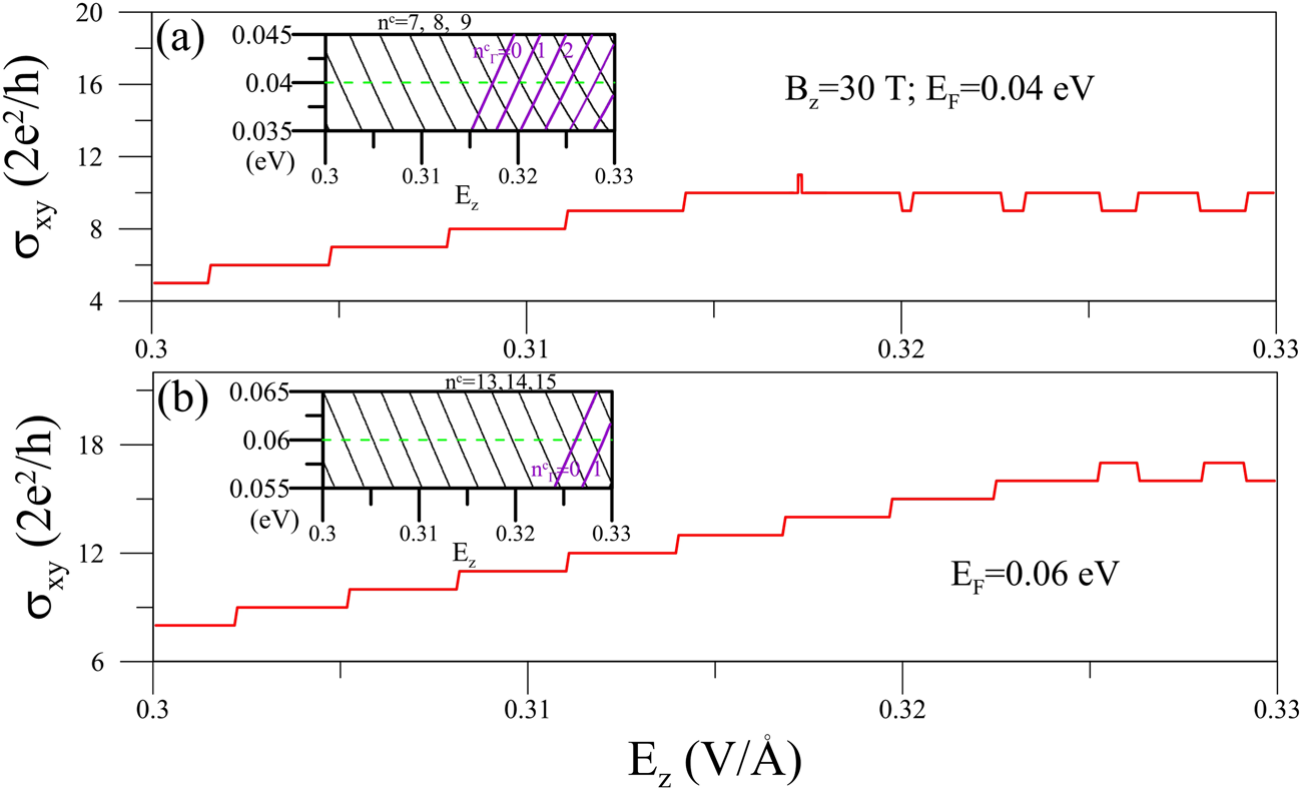
The *E*_*z*_-dependent *σ*_*xy*_ at *B*_*z*_ = 30 T for different *E*_*F*_’s in (**a,b**). The corresponding *E*_*z*_-dependent LL energy spectra are shown in the insets.

The dip and bulging structures appear frequently in *σ*_*xy*_-*E*_*z*_ plots, as shown in Fig. 5(a,b) for different *E*_*F*_’s. The unusual staircase behavior begins when the Γ-group of LLs (in purple color) is formed at *E*_*z*_ > *E*_*z,c*_, as seen in the insets. The higher value of *E*_*F*_ is, the larger *E*_*z,c*_ is needed. Contrary to the results determined by varying *B*_*z*_, the quantum number of the highest occupied LL in each group increases with *E*_*z*_. Therefore, the plateau height is increased/reduced passing a LL from the outer/inner constant-energy loops, leading to the well-like staircases. The widths of these particular structures are strongly governed by the LL-spacings/*B*_*z*_-strength.

**Figure 6:**
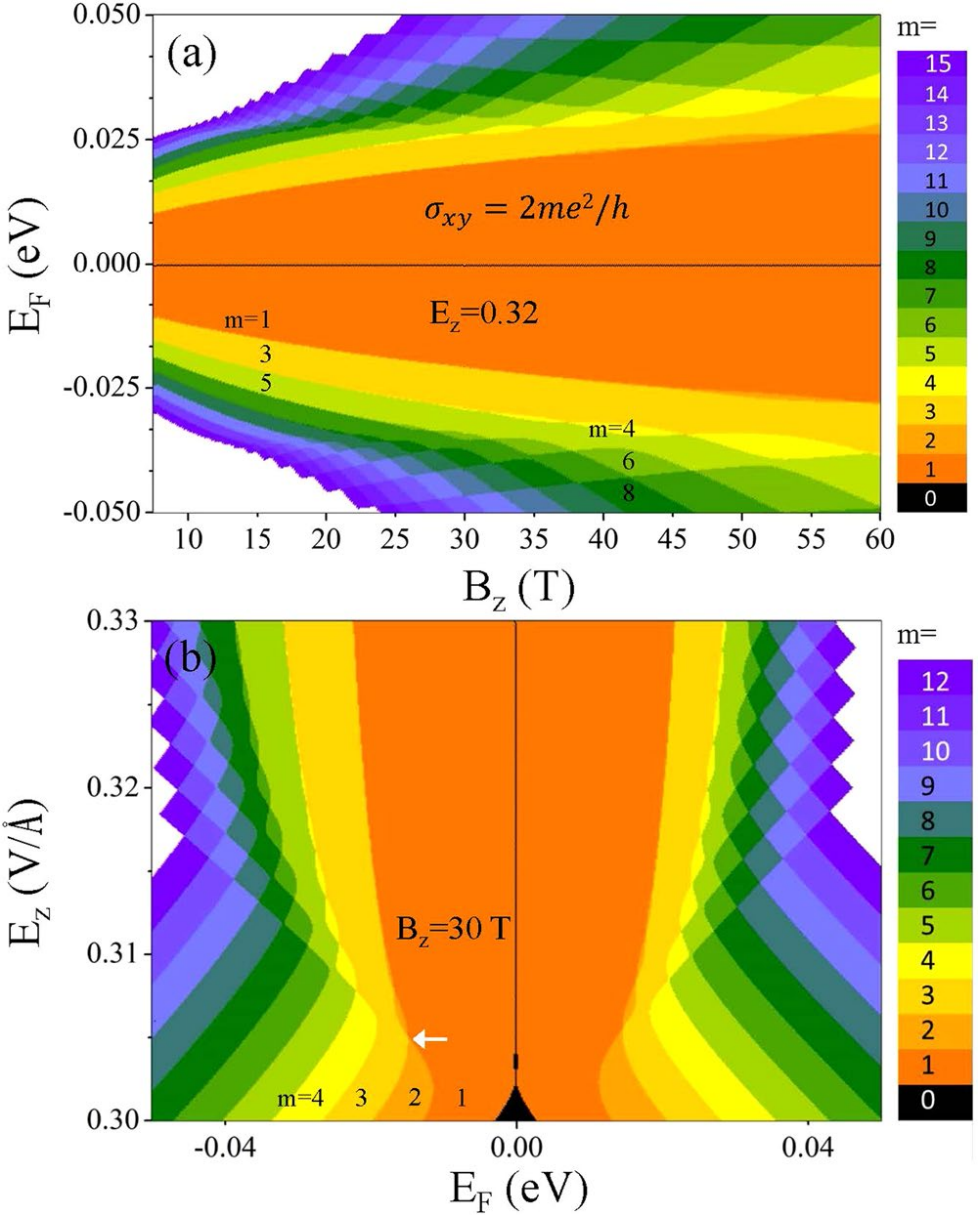
The *E*_*F*_−*B*_*z*_−*σ*_*xy*_ phase diagram in (**a**) and that of *E*_*z*_−*B*_*F*_−*σ*_*xy*_ in (**b**). The color scale represents the value of quantized number *m*, where *m* ≤ 15 in (**a**) and *m* ≤ 12 in (**b**). Also, the white arrow indicates the minimum value of *E*_*z*_ to induce the oscillation mode of even plateaus.

The *E*_*F*_ versus *B*_*z*_ phase diagram for the conductivity (color scale in units of 2*e*^2^/*h*) is helpful to give the whole picture of the feature-rich transport properties. With *E*_*z*_ > *E*_*z,c*_ (Fig. 6(a)), the Hall plateaus appear as colored bands diverging from the charge neutrality point at *B*_*z*_ → 0. For *B*_*z*_ < 10 T, the quantized number *m* around *E*_*F*_ = 0 come in a regular order of odd ones, i.e., *m* = 1,3,5, etc., in correspondence to the D-group of LLs. With the increase of *B*_*z*_, even *m*’s occur in an oscillatory fashion. The larger even value of *m* is, the weaker strength of *B*_*z*_ is required. The oscillation begins around the energy of saddle point (*E*^*c*^ ≈ 0.028 eV; *E*^*v*^ ≈ −0.035 eV), where the D-group LLs are turned into pairs of entangled LLs. The alternating appearance of odd and even *m*’s at low *E*_*F*_ reveals the strong competitive and cooperative relations between the intralayer and interlayer atomic interactions and the Coulomb potentials.

The peculiar change of *m* is also seen in the *E*_*z*_ − *E*_*F*_ − *σ*_*xy*_ phase diagram but with distinct features, as shown in Fig. 6(b). The zero plateau (*m* = 0) exists at *E*_*z*_ < 0.302, where the other higher plateaus obey the usual integer QHE, i.e., *m* = 1,2,3,4, etc. Increasing *E*_*z*_ diminishes the width of zero plateau and raises *m* for a chosen *E*_*F*_. In addition, beyond a critical value of *E*_*z*_ (indicated by the white arrow), the plateaus with an even *m* gradually turn into the oscillatory modes and finally disappear for further stronger *E*_*z*_. At the same time, the half-integer QHE in units of 4*e*^2^/*h* is formed. The oscillatory behavior of *σ*_*xy*_ gets stronger away from *E*_*F*_ = 0, corresponding to the more frequent LL anticrossings/crossings. The two phase plots suggest that one may increase *B*_*z*_/*E*_*z*_ strength to lift/enhance the LL degeneracy, and to lead to the crossover from integer/half-integer to half-integer/integer QHEs. This provides an alternative to possible transport applications.

## Conclusions

The strong electrically tunable energy bands are capable of enriching the quantization properties under a uniform perpendicular magnetic field ${B}_{z}\hat{z}$, such as producing two subgroups of Landau levels (LLs), uniform and non-uniform LL energy spacings, and frequent crossings and anti-crossings. Consequently, the magnetotransport properties of bilayer phosphore cover the integer and half-integer conductivities, the well-like, staircase and composite quantum structures, as well as different step heights and widths. The rich composite effects of intrinsic interactions and external fields are responsible for this. *E*_*F*_ − *B*_*z*_ − *σ*_*xy*_ and *E*_*z*_ − *E*_*F*_ − *σ*_*xy*_ phase plots reveal the transitions between insulator to conductor, integer to half-integer QHEs, and the oscillating quantized numbers.

Our predictions for the diverse quantum conductivities could be experimentally verified by magneto-transport measurements in a dual-gated system, as have been carried out/designed on few-layer graphene^66–68^, and phosphorene^69,70^. Phosphorene is anticipated to be an attractive candidate for designing unconventional thermoelectric devices^39,40^. These results could be further used to understand the magnetothermoelectric transport. In addition, the methodology is readily extended to other main-stream 2D systems, such as few-layer silicene, germanene, tinene, antomonene, bismuthene, etc. We sincerely believe that our systematic study could attract more experimental/theoretical researches on these emergent materials and accelerate their developments for device and energy applications.

## Supplementary information

Supplementary information.
