# Supplementary material for: Anomalous magneto-transport properties of bilayer phosphorene
Source: Sci Rep. 2020 May 6;10:7674. doi: 10.1038/s41598-020-64106-0 (PMC7203127; doi:10.1038/s41598-020-64106-0)
Supplement: Supplementary file 1 — Supplementary information. [file 41598_2020_64106_MOESM1_ESM.pdf]

Anomalous magneto-transport properties of bilayer phosphorene

Jhao-Ying Wu[1,*], Wu-Pei Su[2], Godfrey Gumbs[3]
[1]Center of General Studies, National Kaohsiung University of Science and Technology, Kaohsiung, Taiwan 811
[2]Department of Physics, University of Houston, Houston, Texas
[3]Department of Physics and Astronomy, Hunter College at the City University of New York,
New York, New York 10065, USA

1 The Hamiltonian matrix

Monolayer phosphorene, with a puckered honeycomb structure, has a primitive unit cell containing four phosphorus atoms, as indicated by the dashed yellow lines in Fig. 1(a). Two of the four phosphorus atoms are located on the lower (red circles) or upper (green circles) sublattice sites. The two bond lengths for in-plane and inter-plane P-P connections are $b_1 = 2.24$ Å and $b_2 = 2.22$ Å, respectively. The angle between two in-plane bonds is $\alpha' = 100.7^o$, and that between the inter-plane bond and the x-axis is $\beta' = 69.2^o$. The lattice vectors are $\mathbf{a_1} = 2b_1 sin(\frac{\alpha'}{2})\hat{y}$ and $\mathbf{a_2} = [b_2 cos(\beta') + 2b_1 cos(\frac{\alpha'}{2})]\hat{x}$, as indicated by the yellow arrows in Fig. 1(a). For bilayer phosphorene, AB stacking is the most stable configuration, as revealed in black phosphorus. Such stacking could be regarded as shifting the bottom layer by half of the cell along either $\mathbf{a_1}$ or $\mathbf{a_2}$ direction. As a result, the edge corresponds to the center for the puckered hexagons in two neighboring layers, or vice versa.

The low-lying electronic structure is mainly from the 3p_z-orbital hybridizations. The Hamiltonian of monolayer phosphorene (bilayer AB stacking) is a 4×4 (8×8) Hermitian matrix. The significant atomic interactions include the position-dependent five intralayer h^{ll}_{IJ} and four interlayer $h^{ll'}_{IJ}$ ($l \neq l'$) hopping integrals. The former, as clearly shown by the dashed black arrows in Fig. 1(a), are h^{11}_{12}=3.665 eV, $h^{11}_{21} = -0.055$ eV, $h^{11}_{13} = -0.105$ eV, $h^{11}_{14} = -0.205$ eV, and $h^{11}_{41} = -1.22$ eV. The latter plotted by the dashed yellow arrows (Fig. 1(b)) are $h^{21}_{12} = 0.295$ eV, $h^{21}_{21} = -0.091$ eV, $h^{21}_{24} = -0.151$ eV, and $h^{21}_{42} = 0.273$ eV. The matrix elements related to the intralayer interactions are given by:

$$
\begin{aligned}
\langle B^l_{I\mathbf{k}}|H|A^{l'}_{J\mathbf{k}}\rangle &= \langle A^l_{I\mathbf{k}}|H|B^{l'}_{J\mathbf{k}}\rangle = (h^{11}_{12}t'_2 + h^{11}_{21}t'_5)\delta_{I+1,J}\delta_{l,l'}, \\
\langle B^l_{I\mathbf{k}}|H|A^{l'}_{J\mathbf{k}}\rangle &= \langle A^l_{I\mathbf{k}}|H|B^{l'}_{J\mathbf{k}}\rangle = (h^{11}_{13}t'_4 + h^{11}_{13}tt'_4)\delta_{I+2,J}\delta_{l,l'}, \\
\langle B^l_{I\mathbf{k}}|H|B^{l'}_{J\mathbf{k}}\rangle &= (h^{11}_{41}tt'_1 + h^{11}_{14}t'_3)\delta_{I+3,J}\delta_{l,l'}, \\
\langle B^l_{I\mathbf{k}}|H|B^{l'}_{J\mathbf{k}}\rangle &= (h^{11}_{41}t'_1 + h^{11}_{14}t'_3)\delta_{I,J+3}\delta_{l,l'}, \\
\langle A^l_{I\mathbf{k}}|H|A^{l'}_{J\mathbf{k}}\rangle &= (h^{11}_{41}tt'_1 + h^{11}_{14}t'_3)\delta_{I,J-1}\delta_{l,l'}, \\
\langle A^l_{I\mathbf{k}}|H|A^{l'}_{J\mathbf{k}}\rangle &= (h^{11}_{41}tt'_1 + h^{11}_{14}tt'_3)\delta_{I+1,J}\delta_{l,l'},
\end{aligned} \tag{1}
$$

where $I = 1,2,3;4$. The position-dependent phase terms for the intralayer hoppings are written below:

$$
\begin{aligned}
t'_1 &= \exp i(k_x d_{1x} + k_y d_{1y}) + \exp i(k_x d_{1x} - k_y d_{1y}), \\
tt'_1 &= \exp i(-k_x d_{1x} + k_y d_{1y}) + \exp i(-k_x d_{1x} - k_y d_{1y}), \\
t'_2 &= \exp i(k_x d_{2x}), \\
t'_3 &= \exp i(k_x d_{3x} + k_y d_{1y}) + \exp i(k_x d_{3x} - k_y d_{1y}), \\
tt'_3 &= \exp i(-k_x d_{3x} + k_y d_{1y}) + \exp i(-k_x d_{3x} - k_y d_{1y}), \\
t'_4 &= \exp i(k_x d_{4x} + k_y d_{1y}) + \exp i(k_x d_{4x} - k_y d_{1y}), \\
tt'_4 &= \exp i(-k_x d_{4x} + k_y d_{1y}) + \exp i(-k_x d_{4x} - k_y d_{1y}), \\
t'_5 &= \exp i(-k_x d_{5x}),
\end{aligned} \tag{2}
$$

where d_{1x-5x} (d_{1y}) is the corresponding distance along the x (y)-axis between the interacting lattice sites. These distance

parameters are expressed below:

$$\begin{aligned}
d_{1x} &= b_1 cos(\frac{\alpha'}{2}),\\
d_{2x} &= b_2 cos\beta',\\
d_{3x} &= b_1 cos(\frac{\alpha'}{2})+2b_2 cos\beta',\\
d_{4x} &= b_1 cos(\frac{\alpha'}{2})+b_2 cos\beta',\\
d_{5x} &= 2b_1 cos(\frac{\alpha'}{2})+b_2 cos\beta';\\
d_{1y} &= b_1 sin(\frac{\alpha'}{2}).
\end{aligned} \tag{3}$$

As for interlayer interactions, the independent Hamiltonian matrix elements are:

$$\begin{aligned}
\langle A^l_{I\mathbf{k}}|H|B^{l'}_{J\mathbf{k}}\rangle &= (h^{21}_{42}tt''_2+h^{21}_{42}t''_2+h^{21}_{24}tt''_3+h^{21}_{24}t''_3)\delta_{I+1,J}\delta_{l,l'+1},\\
\langle A^l_{I\mathbf{k}}|H|B^{l'}_{J\mathbf{k}}\rangle &= (h^{21}_{42}tt''_2+h^{21}_{42}t''_2+h^{21}_{24}tt''_3+h^{21}_{24}t''_3)\delta_{I,J+2}\delta_{l,l'+1},\\
\langle A^l_{I\mathbf{k}}|H|B^{l'}_{J\mathbf{k}}\rangle &= (h^{21}_{12}tt''_1+h^{21}_{21}t''_4)\delta_{I,J+1}\delta_{l,l'+1};\\
\langle A^l_{I\mathbf{k}}|H|B^{l'}_{J\mathbf{k}}\rangle &= (h^{21}_{12}t''_1+h^{21}_{21}tt''_4)\delta_{I+1,J}\delta_{l,l'+1}.
\end{aligned} \tag{4}$$

The position-dependent phase terms for the interlayer hoppings are as follows:

$$\begin{aligned}
t''_1 &= \exp i(k_x d_{2x}+k_y d_{1y})+\exp i(k_x d_{2x}-k_y d_{1y}),\\
tt''_1 &= \exp i(-k_x d_{2x}+k_y d_{1y})+\exp i(-k_x d_{2x}-k_y d_{1y}),\\
t''_2 &= \exp i(k_x d_{4x}),\\
tt''_2 &= \exp i(-k_x d_{4x}),\\
t''_3 &= \exp i(k_x d_{4x}+k_y 2d_{1y})+\exp i(k_x d_{4x}-k_y 2d_{1y}),\\
tt''_3 &= \exp i(-k_x d_{4x}+k_y 2d_{1y})+\exp i(-k_x d_{4x}-k_y 2d_{1y}),\\
t''_4 &= \exp i(k_x d_{5x}+k_y d_{1y})+\exp i(k_x t_{5x}-k_y d_{1y});\\
tt''_4 &= \exp i(-k_x d_{5x}+k_y d_{1y})+\exp i(-k_x d_{5x}-k_y d_{1y}).
\end{aligned} \tag{5}$$

When monolayer and bilayer phosphorene systems exist in a perpendicular magnetic field, the magnetic flux through a puckered hexagon is $\Phi = a_1 a_2 B_z/2$. The vector potential, $\mathbf{A} = (B_z x)\hat{y}$, leads to a new period along $\hat{x}$ and thus an enlarged rectangular unit cell with $4R_B = 4\Phi_0/\Phi$ atoms in monolayer, as illustrated in Fig. 1(c). The reduced first Brilloun zone has an area of $4\pi^2/a_1 a_2 R_B$. The magnetic matrix elements can be obtained by adding the extra Peierls phases, as given below:

(I) intralayer interactions:

$$\begin{aligned}
tt'_{1,I=odd} &= \exp i(-k_x d_{1x}+k_y d_{1y}+\pi\frac{\Phi}{\Phi_0}(I-1+\frac{2d_{2x}-d_{3x}}{d_{4x}}))\\
&\quad +\exp i(k_x d_{1x}-k_y d_{1y}-\pi\frac{\Phi}{\Phi_0}(I-1+\frac{2d_{2x}-d_{3x}}{2d_{4x}})),\\
t'_{3,I=odd} &= \exp i(k_x d_{3x}+k_y d_{1y}+\pi\frac{\Phi}{\Phi_0}(I-1+\frac{2d_{2x}+d_{1x}}{2d_{4x}}))\\
&\quad +\exp i(k_x d_{3x}-k_y d_{1y}+\pi\frac{\Phi}{\Phi_0}(I-1+\frac{2d_{2x}+d_{1x}}{2d_{4x}})),\\
tt'_{3,I=odd} &= \exp i(-k_x d_{3x}+k_y d_{1y}+\pi\frac{\Phi}{\Phi_0}(I-1-\frac{2d_{2x}+d_{1x}}{2d_{4x}}))\\
&\quad +\exp i(-k_x d_{3x}-k_y d_{1y}-\pi\frac{\Phi}{\Phi_0}(I-1-\frac{2d_{2x}+d_{1x}}{2d_{4x}})),\\
t'_{4,I=odd} &= \exp i(k_x d_{4x}+k_y d_{1y}+\pi\frac{\Phi}{\Phi_0}(I-\frac{1}{2}))\\
&\quad +\exp i(k_x d_{4x}-k_y d_{1y}-\pi\frac{\Phi}{\Phi_0}(I-\frac{1}{2})),
\end{aligned} \tag{6}$$

$$
\begin{aligned}
tt'_{4,I=odd} &= \exp i(-k_x d_{4x} + k_y d_{1y} + \pi \frac{\Phi}{\Phi_0}(I - \frac{3}{2})) \\
&\quad + \exp i(-k_x d_{4x} - k_y d_{1y} - \pi \frac{\Phi}{\Phi_0}(I - \frac{3}{2})), \\
t'_{4,I=even} &= \exp i(k_x d_{4x} + k_y d_{1y} + \pi \frac{\Phi}{\Phi_0}(I - 1 + \frac{2d_{2x} + d_{4x}}{2d_{4x}})) \\
&\quad + \exp i(k_x d_{4x} - k_y d_{1y} - \pi \frac{\Phi}{\Phi_0}(I - 1 + \frac{2d_{2x} + d_{4x}}{2d_{4x}})), \\
tt'_{4,I=even} &= \exp i(-k_x d_{4x} + k_y d_{1y} + \pi \frac{\Phi}{\Phi_0}(I - 1 - \frac{2d_{2x} + d_{4x}}{2d_{4x}})) \\
&\quad + \exp i(-k_x d_{4x} - k_y d_{1y} - \pi \frac{\Phi}{\Phi_0}(I - 1 - \frac{2d_{2x} + d_{4x}}{2d_{4x}})), \\
t'_{1,I=even} &= \exp i(k_x d_{1x} + k_y d_{1y} + \pi \frac{\Phi}{\Phi_0}(I - 1 + \frac{2d_{2x} + d_{1x}}{2d_{4x}})) \\
&\quad + \exp i(k_x d_{1x} - k_y d_{1y} - \pi \frac{\Phi}{\Phi_0}(I - 1 + \frac{2d_{2x} + d_{1x}}{2d_{4x}})); \\
tt'_{3,I=even} &= \exp i(-k_x d_{3x} + k_y d_{1y} + \pi \frac{\Phi}{\Phi_0}(I - 1 + \frac{2d_{2x} - d_{3x}}{2d_{4x}})) \\
&\quad + \exp i(-k_x d_{3x} - k_y d_{3y} - \pi \frac{\Phi}{\Phi_0}(I - 1 + \frac{2d_{2x} - d_{3x}}{2d_{4x}})). \qquad (7)
\end{aligned}
$$

(II) interlayer interactions:

$$
\begin{aligned}
t''_{1,I=odd} &= \exp i(k_x d_{2x} + k_y d_{1y} + \pi\frac{\Phi}{\Phi_0}(I-1+\frac{d_{2x}}{2d_{4x}})) \\
&\quad +\exp i(k_x d_{2x} - k_y d_{1y} - \pi\frac{\Phi}{\Phi_0}(I-1+\frac{d_{2x}}{2d_{4x}})), \\
tt''_{1,I=odd} &= \exp i(-k_x d_{2x} + k_y d_{1y} + \pi\frac{\Phi}{\Phi_0}(I-1-\frac{d_{2x}}{2d_{4x}})) \\
&\quad +\exp i(-k_x d_{2x} - k_y d_{1y} - \pi\frac{\Phi}{\Phi_0}(I-1-\frac{d_{2x}}{2d_{4x}})), \\
t''_{1,I=even} &= \exp i(k_x d_{2x} + k_y d_{1y} + \pi\frac{\Phi}{\Phi_0}(I-1-\frac{d_{2x}}{2d_{4x}})) \\
&\quad +\exp i(k_x d_{2x} - k_y d_{1y} - \pi\frac{\Phi}{\Phi_0}(I-1-\frac{d_{2x}}{2d_{4x}})), \\
tt''_{1,I=even} &= \exp i(-k_x d_{2x} + k_y d_{1y} + \pi\frac{\Phi}{\Phi_0}(I-1+\frac{d_{2x}}{2d_{4x}})) \\
&\quad +\exp i(-k_x d_{2x} - k_y d_{1y} - \pi\frac{\Phi}{\Phi_0}(I-1+\frac{d_{2x}}{2d_{4x}})), \\
t''_{3,I=odd} &= \exp i(k_x d_{4x} + k_y 2d_{1y} + 2\pi\frac{\Phi}{\Phi_0}(I-\frac{1}{2})) \\
&\quad +\exp i(k_x d_{4x} - k_y 2d_{1y} - 2\pi\frac{\Phi}{\Phi_0}(I-\frac{1}{2})), \\
tt''_{3,I=odd} &= \exp i(-k_x d_{4x} + k_y 2d_{1y} + 2\pi\frac{\Phi}{\Phi_0}(I-\frac{3}{2})) \\
&\quad +\exp i(-k_x d_{4x} - k_y 2d_{1y} - 2\pi\frac{\Phi}{\Phi_0}(I-\frac{3}{2})), \\
t''_{3,I=even} &= \exp i(k_x d_{4x} + k_y 2d_{1y} + 2\pi\frac{\Phi}{\Phi_0}(I-1+\frac{2d_{2x}+d_{4x}}{2d_{4x}})) \\
&\quad +\exp i(k_x d_{4x} - k_y 2d_{1y} - 2\pi\frac{\Phi}{\Phi_0}(I-1+\frac{2d_{2x}+d_{4x}}{2d_{4x}})), \\
tt''_{3,I=even} &= \exp i(-k_x d_{4x} + k_y 2d_{1y} + 2\pi\frac{\Phi}{\Phi_0}(I-1-\frac{2d_{2x}+d_{4x}}{2d_{4x}})) \\
&\quad +\exp i(-k_x d_{4x} - k_y 2d_{1y} - 2\pi\frac{\Phi}{\Phi_0}(I-1-\frac{2d_{2x}+d_{4x}}{2d_{4x}})), \\
t''_{4,I=odd} &= \exp i(k_x d_{5x} + k_y d_{1y} + \pi\frac{\Phi}{\Phi_0}(I-1-\frac{d_{5x}}{2d_{4x}})) \\
&\quad +\exp i(k_x d_{5x} - k_y d_{1y} - \pi\frac{\Phi}{\Phi_0}(I-1-\frac{d_{5x}}{2d_{4x}})), \\
tt''_{4,I=odd} &= \exp i(-k_x d_{5x} + k_y d_{1y} + \pi\frac{\Phi}{\Phi_0}(I-1+\frac{d_{5x}}{2d_{4x}})) \\
&\quad +\exp i(-k_x d_{5x} - k_y d_{1y} - \pi\frac{\Phi}{\Phi_0}(I-1+\frac{d_{5x}}{2d_{4x}})),
\end{aligned}
\tag{8}
$$

$$
\begin{aligned}
t''_{4,I=even} &= \exp i(k_x d_{5x} + k_y d_{1y} + \pi\frac{\Phi}{\Phi_0}(I-1+\frac{d_{2x}}{2d_{4x}})) \\
&\quad +\exp i(k_x d_{5x} - k_y d_{1y} - \pi\frac{\Phi}{\Phi_0}(I-1+\frac{d_{2x}}{2d_{4x}})), \\
tt''_{4,I=even} &= \exp i(-k_x d_{5x} + k_y d_{1y} + \pi\frac{\Phi}{\Phi_0}(I-1-\frac{d_{2x}}{2d_{4x}})) \\
&\quad +\exp i(-k_x d_{5x} - k_y d_{1y} - \pi\frac{\Phi}{\Phi_0}(I-1-\frac{d_{2x}}{2d_{4x}})),
\end{aligned}
\tag{9}
$$

where $I = 1, 2, 3.....4R_B$. It should be noticed that the magnetic Hamiltonian matrix elements are real numbers for the ($k_x =$

$0, k_y$) LL states.

2 Subgroups of low-lying LLs

The two subgroups of the low-lying LLs discussed in this paper is created by $E_z > E_{z,c}$, where $E_{z,c}$ is proportional to B_z. That is, when the energies of $n^{c,v} = 1$ LLs are larger/smaller than those of the extreme points at the Γ point ($E^c \approx$0.048 eV; $E^v \approx$-0.055 eV), only one group of LLs is observed, as clearly illustrated in Figure S1.

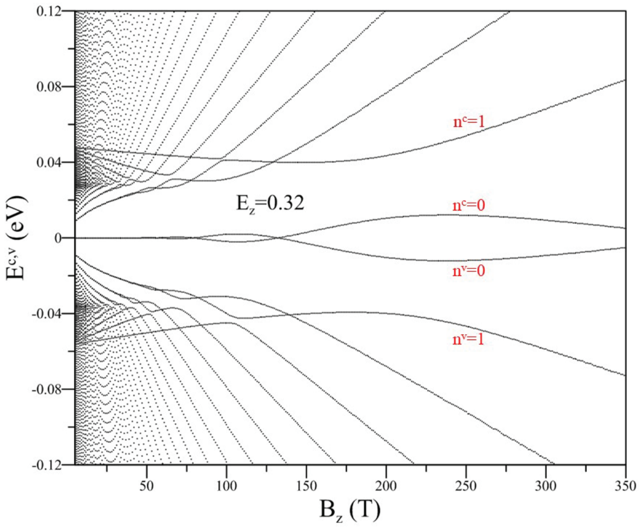

Figure S1. The B_z-dependent LL spectrum at $E_z = 0.32$.
